# A case of Frégoli syndrome following 24-hour levodopa-carbidopa intestinal gel infusion in a patient with Parkinson’s disease undergoing STN-DBS

**DOI:** 10.1016/j.prdoa.2025.100364

**Published:** 2025-06-30

**Authors:** Shunsuke Ogata, Katsuo Kimura, Katsuya Abe, Yumeko Urago, Noriko Hayashi, Misako Kunii, Naohisa Ueda, Fumiaki Tanaka

**Affiliations:** a Department of Neurology, Yokohama City University Medical Center. 4-57, Urafune-cho, Minami-ku, Yokohama 232-0024, Japan; b Department of Neurology and Stroke Medicine, Yokohama City University Graduate School of Medicine, 3-9, Fukuura, Kanazawa-ku, Yokohama 236-0004, Japan

**Keywords:** Parkinson’s disease, Levodopa-carbidopa intestinal gel (LCIG), Psychosis, Frégoli syndrome, Continuous dopaminergic stimulation

Delusional misidentification syndromes (DMSs) are rare psychiatric disorders characterized by persistent delusions involving people, place, or object misidentification. In Frégoli syndrome, a particularly rare subtype, patients believe that familiar individuals disguise themselves to deceive or monitor them [1].

We report a case of Frégoli syndrome in a 50-year-old male with Parkinson’s disease (PD) treated with subthalamic nucleus deep brain stimulation (STN-DBS); the condition developed after a self-initiating 24-hour intra-jejunal levodopa-carbidopa intestinal gel (LCIG) infusion, exceeding Japan’s 16-hour approved limit.

The patient’s diagnosis with PD at age 37 years involved left-dominant akinesia; he developed auditory hallucinations, persecutory delusions, and gait disturbances due to wearing-off, following initial response to levodopa; he also developed levodopa-induced dyskinesia. At 42 years, he underwent STN-DBS targeting the dorsolateral STN. Motor symptoms improved temporarily without psychiatric complications; however, despite DBS adjustments (amplitude: 2.3 mA left, 2.5 mA right; pulse width: 30 µs bilaterally; frequency: 84 Hz left, 119 Hz right), symptom fluctuations persisted and were managed with oral levodopa.

At 46 years, he commenced a 16-hour LCIG infusion (864 mg/day) with rotigotine (6 mg/day) to alleviate nocturnal motor symptoms. STN-DBS was maintained throughout the LCIG therapy. Initially, motor symptoms improved without psychiatric symptoms; however, he became levodopa-dependent and self-administered extra boluses, by extending his LCIG infusion to 24 h and the dose to 1056 mg/day, without medical supervision. Two months before admission, he developed poverty delusions, self-doubt, and accusatory auditory hallucinations. He acted on these delusions, walking barefoot outside, intermittently stopping LCIG, and self-doubling his rotigotine dose (12 mg/day), leading to erratic medication use.

Upon admission, he could not walk independently due to wearing-off. His Mini-Mental State Examination score of 30/30 suggested preserved overall cognition, although he appeared despondent, with reduced food intake, financial distress, self-hatred, and suicidal ideation. He was admitted to the psychiatric ward due to self-harm risk. Bolus dosing was discontinued; LCIG was increased to 1248 mg/day to prioritize motor symptom management. Psychiatric symptoms were treated with quetiapine, minimally affecting motor function (12.5 mg/day, titrated to 75 mg/day over 10 days), and valproic acid (400 mg/day from day 10) for mood stabilization. Rotigotine was discontinued due to suspected psychiatric effects. However, his condition worsened, and he developed Frégoli syndrome [1], believing that a disliked acquaintance had disguised themselves as a nurse or patient to monitor and deceive him. Persecutory delusions and self-harm, such as attempting to injure himself with the LCIG pump strap, persisted.

On hospital day 21, LCIG infusion was shortened from 24 to 16 h, maintaining the 1248 mg/day dose and antipsychotic regimen. After 1 week, delusions and hallucinations markedly improved, and food intake normalized. Motor fluctuations subsided and stabilized; 2 weeks later, he was discharged, advised against unauthorized LCIG adjustments, and adherence was emphasized. Antipsychotics were tapered post-discharge, with no neuropsychiatric symptoms or levodopa dependency for over 10 months.

This case represents a rare instance of PD with Frégoli syndrome, possibly associated with the 24-hour LCIG infusion. Though typically seen in schizophrenia or bipolar disorder, it can also arise from stroke or brain injury [1]. Despite known dopaminergic side effects in PD like hallucinations and impulse control disorders, DMSs like Frégoli syndrome are rare, with only one prior case linked to oral levodopa [2]. The pathogenesis of Frégoli syndrome remains unclear, though excessive mesolimbic dopaminergic activity may play a role [3]. In secondary psychiatric disorders, it is often associated with the right frontal lobe [1]; 18F-fluorodeoxyglucose-PET imaging shows right striatal hypermetabolism [4].

The patient also exhibited dopamine dysregulation syndrome (DDS), characterized by levodopa dependency and compulsive self-dosing [5], which led to 24-hour LCIG use and triggered DMS. The mesolimbic circuitry, involved in addiction and behavioral reinforcement, contributes to DDS, suggesting shared pathophysiology with DMS; indeed, DDS is often followed by delusions [5].

Continuous dopaminergic stimulation is standard in advanced PD to reduce motor complications, with LCIG being a well-established approach. Although 16-hour infusions are approved in Japan, 24-hour regimens have been trialed for nocturnal symptoms, sleep disturbances, refractory dyskinesia, and gait freezing [6]. In one study, among 12 patients on 24-hour LCIG, 3 reported psychiatric symptoms, suggesting longer exposure increases neuropsychiatric risk [6]. One patient improved after reverting to the 16-hour LCIG infusion; others improved with reduced nocturnal dosing. Another patient with PD and Frégoli syndrome improved following oral levodopa dose reduction [2], reinforcing the role of dopaminergic overstimulation. Uniquely, as our case showed, discontinuing the nighttime LCIG infusion without reducing the total daily dose resolves psychiatric symptoms.

The 24-hour LCIG infusion disrupts normal diurnal dopamine rhythms, causing excess nighttime dopamine when levels are typically low. Additionally, dopaminergic terminal degeneration in PD reduces buffering capacity, causing significant striatal dopamine fluctuations. Thus, iatrogenic nocturnal dopamine dysregulation—rather than total daily dose—may drive hallucinations and delusions in PD. This hypothesis is supported by a foslevodopa-foscarbidopa trial, a 24-hour subcutaneous levodopa prodrug [7], where 15 % of patients on continuous infusion had psychiatric events vs. 3 % on oral levodopa-carbidopa, despite similar daily doses (approximately 1000 mg/day), suggesting constant delivery caused sustained nighttime dopamine elevation.

This report has several limitations. First, possible confounders, including STN-DBS, medication misuse, and antipsychotic use, cannot be ruled out as contributors to the psychiatric symptoms. However, DBS uses low-intensity stimulation targeting the dorsolateral STN, which is less associated with psychiatric symptoms. Moreover, the settings remained stable, and no psychiatric symptoms occurred over 8 years post-implantation, suggesting limited DBS involvement. Symptoms persisted despite stopping LCIG bolus and rotigotine, and antipsychotic doses remained stable, making these unlikely causes; reducing LCIG duration highlighted its role. Second, the lack of neuroimaging studies like 18F-fluorodeoxyglucose-PET limits our understanding of the underlying neurobiological mechanisms of Frégoli syndrome. Finally, objective measures for motor and psychiatric symptoms were not utilized.

This case highlights the need to educate patients on balancing motor control and psychiatric risks with LCIG and the importance of appropriate use and avoidance of unauthorized adjustments. Current LCIG protocols cannot record bolus frequency or infusion duration; thus, regular clinical monitoring is crucial. Prolonged nighttime dopaminergic stimulation may cause psychiatric complications, including Frégoli syndrome, warranting careful management.

## Ethical approval and patient consent

Written informed consent was obtained from the patient following institutional and ethical guidelines.

Funding

The authors declare that no financial support was received for the research, authorship, or publication of this article.

## CRediT authorship contribution statement

**Shunsuke Ogata:** Writing – review & editing, Writing – original draft, Project administration, Investigation, Formal analysis, Data curation, Conceptualization. **Katsuo Kimura:** Writing – review & editing, Project administration, Methodology, Investigation, Formal analysis, Data curation, Conceptualization. **Katsuya Abe:** Writing – review & editing. **Yumeko Urago:** Writing – review & editing. **Noriko Hayashi:** Writing – review & editing. **Misako Kunii:** Writing – review & editing. **Naohisa Ueda:** Writing – review & editing, Supervision. **Fumiaki Tanaka:** Writing – review & editing, Supervision, Conceptualization.

## Declaration of competing interest

Katsuo Kimura reports grants and lecture fees from Kyowa Kirin Co. Ltd., grants from Novartis AG, and lecture fees from Medtronic Japan Co., Ltd., Boston Scientific Corporation, Ono Pharmaceutical Co., Ltd., Otsuka Pharmaceutical Co., Ltd., AbbVie GK, Eisai Co., Ltd., Takeda Pharmaceutical Co., Ltd., and Sumitomo Pharma Co., Ltd. Katsuya Abe and Shunsuke Ogata have received lecture fees from AbbVie GK. Other authors report no disclosures.
